# Takotsubo Cardiomyopathy Associated with Acute COVID-19 Pneumonia

**DOI:** 10.7759/cureus.44483

**Published:** 2023-08-31

**Authors:** Domenic Grosso, Shahzad Akbar, Melisa Esposito, Alex Card

**Affiliations:** 1 Internal Medicine, Kettering Health Main Campus, Kettering, USA

**Keywords:** undifferentiated shock, covid-19 pneumonia, covid-19 associated takotsubo cardiomyopathy, cardiogenic shock, sars-cov-2 (severe acute respiratory syndrome coronavirus -2)

## Abstract

Cardiogenic shock carries a high burden of morbidity and mortality because of inadequate tissue perfusion leading to end-stage multi-organ damage. The initial work-up includes a pertinent and thorough history and physical examination to identify possible cardiac and noncardiac etiologies. The following case describes a patient presenting with symptomatic acute COVID-19 (SARS-CoV-2) pneumonia with initial findings consistent with cardiogenic shock. SARS-CoV-2 pneumonia has been associated with multiple cardiac manifestations including myocarditis, heart failure, myocardial infarction, and Takotsubo cardiomyopathy. This patient was treated with conservative medical management and had complete clinical recovery and normal cardiac angiography weeks after their initial presentation. This clinical scenario highlights the significance of a broad differential and extensive work-up when faced with a patient presenting with cardiogenic shock.

## Introduction

Takotsubo cardiomyopathy (TTC) is an acute reduction in left ventricular ejection fraction (LVEF) due to hypokinesis and ballooning of the cardiac apex with myocardial stunning. This was first described in the literature as early as 1982 [1-3]. In patients with symptoms consistent with acute coronary syndrome (ACS) or an elevated troponin, the incidence of TTC is as high as 1-2% [2]. The influx of a large quantity of catecholamines into cardiac myocytes is believed to be the underlying pathogenesis leading to cardiac dysfunction seen in TTC. This catecholamine surge can have a diverse range of underlying etiologies and is typically associated with emotional stress, infection, or acute illness.

COVID-19, the novel coronavirus variant causing SARS-CoV-2, can present with symptoms including dyspnea, fever, chills, nausea, anosmia, and respiratory decompensation. Although pulmonary manifestations are typical, rarely, COVID-19 may cause myocardial injury, leading to myocarditis, heart failure, myocardial infarction, and even TTC [4].

## Case presentation

A 72-year-old female with no significant past medical history presented to the hospital with a one-week history of shortness of breath, dry, nonproductive cough, and bilateral pedal edema. She attempted over-the-counter therapy with no significant symptomatic improvement. She denied any known sick contacts or occupational exposure. Despite the patient's age, she reported no known past medical history. She did have a 30-pack-year smoking history but denied a personal or family history of cardiovascular disease.

On initial evaluation, she was ill-appearing and exhibited mild respiratory distress with tachypnea and accessory muscle use. Vitals signs were significant for blood pressure 94/47, heart rate 107 beats per minute, temperature 98.4 Fahrenheit, respiratory rate 25 breaths per minute, and oxygen saturation 89%. Cardiac auscultation was significant for tachycardia, regular rhythm, normal S1 and S2, and no appreciable murmurs, rubs, or gallops. Pulmonary auscultations revealed fine bibasilar crackles. Physical examination was otherwise noncontributory.

**Figure 1:**
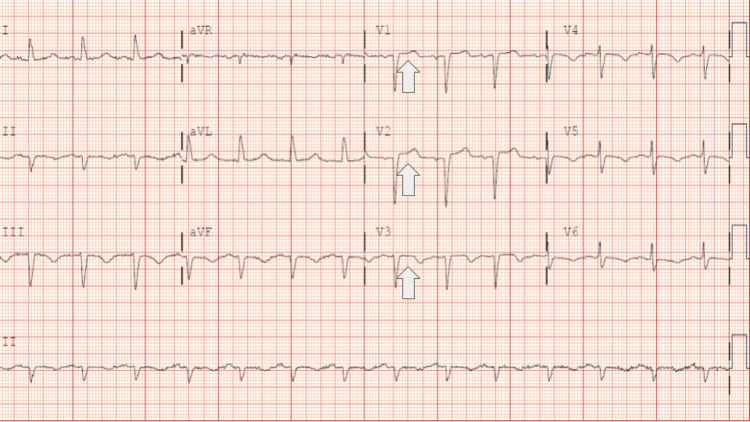
EKG demonstrating anterior Q waves with newly diagnosed incomplete left bundle branch block with secondary ST changes

**Figure 2:**
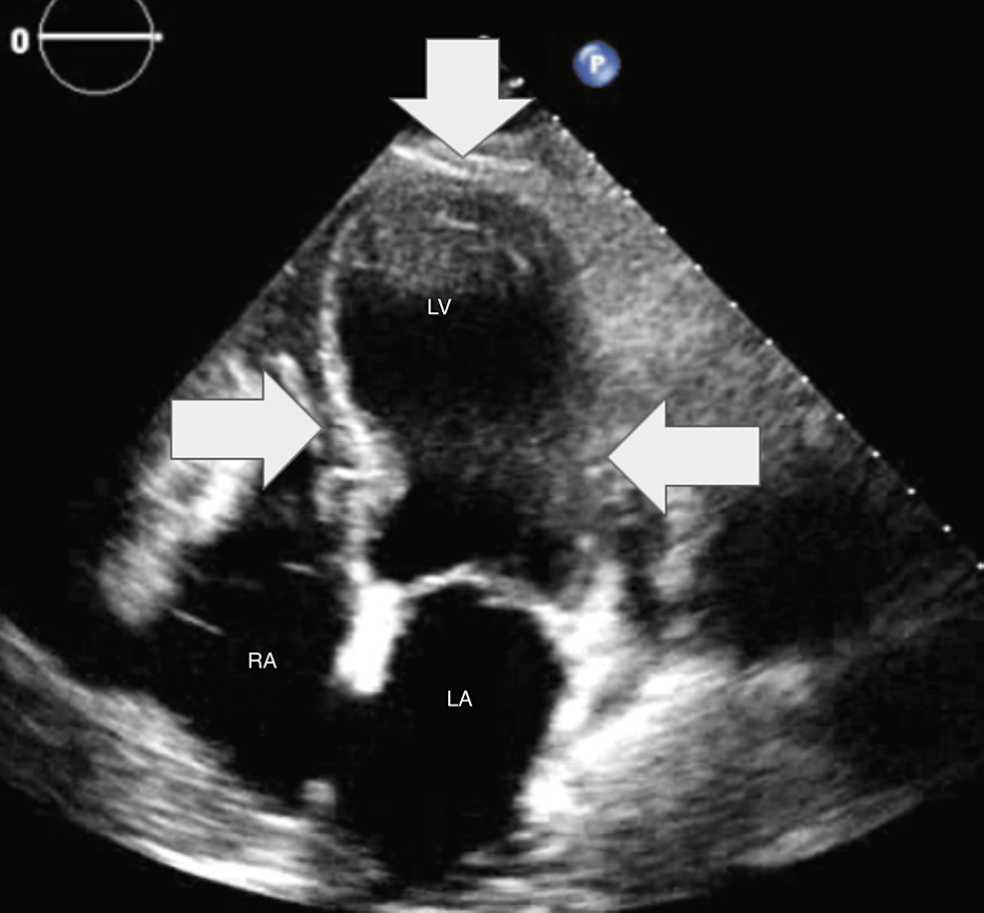
Apical four-chamber transthoracic echocardiogram (end-systolic frame) demonstrating apical ballooning with hypokinesis/akinesis and reduced ejection fraction LV: left ventricle, LA: left atrium, RA: right atrium

**Figure 3:**
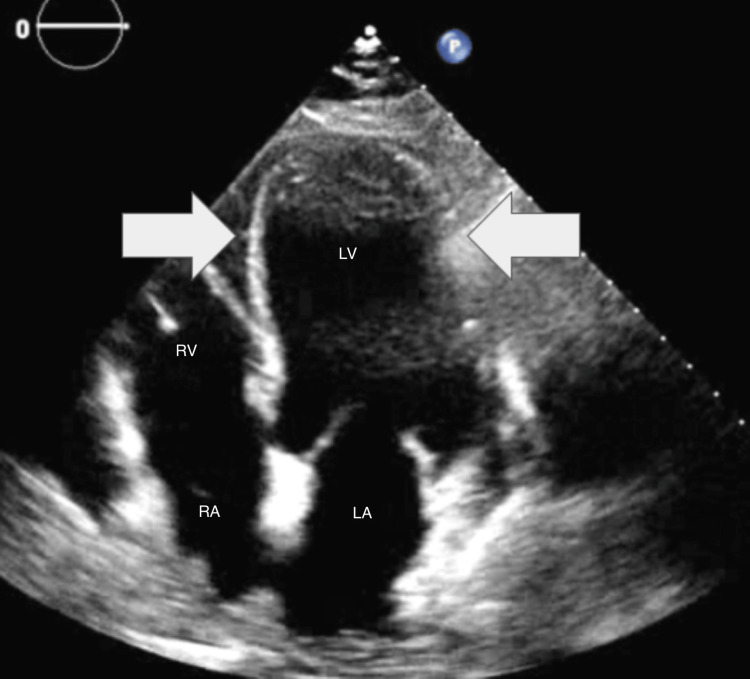
Apical four-chamber transthoracic echocardiogram (end-diastole frame) demonstrating apical ballooning and hypokinesis/akinesis LV: left ventricle, LA: left atrium, RA: right atrium, RV: right ventricle

Initial laboratory studies demonstrated mild leukocytosis with a white blood cell count of 12.2 K/uL (4.0-10.5 K/uL), CRP 28.90 mg/L (<5.00 mg/L), creatinine 1.4 mg/dL (0.6-1.2 mg/dL), and glomerular filtration rate 54 mL/min indicating acute kidney injury stage I. Serial high sensitivity cardiac troponin I was significantly elevated (2.97) (<0.04 ng/mL) and uptrending (3.78) after six hours. EKG demonstrated anterior Q waves with a newly diagnosed incomplete left bundle branch block with secondary ST changes (Figure 1). A CT with angiography of the chest was performed, which was negative for acute pulmonary embolism but demonstrated diffuse patchy airspace disease with prominent mediastinal lymph nodes. Finally, bedside echocardiogram demonstrated apical ballooning and hypokinesis and akinesis of the apical and anteroseptal myocardium with severely reduced LVEF (28%), no mitral or tricuspid regurgitation, consistent with TTC (Figures 2-3). Of note, the patient denied recent significant life stressors, including family or close friend deaths or known illnesses.

The underlying etiology of TTC was not yet evident. Although it was deemed somewhat less likely given the lack of chest pain, overall clinical presentation, and wall motion abnormalities not consistent with those expected from left anterior descending artery (LAD) territory, the differential diagnosis included LAD ischemia/infarction. Cardiology was consulted for further evaluation and assistance with management and recommended therapeutic Lovenox, 1 mg/body mass kg for at least 48 hours, given the possibility of underlying ACS. She was also managed supportively with intravenous fluid resuscitation as she received two liters of normal saline upon presentation.

Despite adequate resuscitation, the patient remained hypotensive, requiring transfer to the ICU for shock of unknown origin as well as acute hypoxic respiratory failure requiring 4 liters of supplemental oxygen via nasal cannula. She was initiated on norepinephrine 5 mcg/hr, empiric broad-spectrum antibiotics with vancomycin and piperacillin-tazobactam, and continued anticoagulation with therapeutic enoxaparin 1 mg/body mass kg two times daily.

Further evaluation included SARS-CoV-2 screening via rapid antigen testing, which was positive for COVID-19. Findings were subsequently confirmed by polymerase chain reaction testing, and the patient was transferred to an isolated unit. The patient was then started on Decadron and Remdesivir. She was able to be weaned off of vasopressors and transferred out of the ICU after two days. Vancomycin and piperacillin-tazobactam were discontinued, but in light of the severe illness, she was transitioned to ceftriaxone to complete a seven-day course of empiric antibiotics. She was also given supportive care, and her respiratory status ultimately improved over the course of her 10-day hospitalization. Follow-up troponin I downtrended (0.290). Follow-up creatinine remained stable (1.0) at the time of discharge. Cardiology started the patient on metoprolol-XL 12.5 mg daily, lisinopril 2.5 mg daily, aspirin 81 mg daily, and atorvastatin 40 mg daily. As the patient showed substantial clinical improvement with supportive measures, she was recommended to follow up with her primary care physician and cardiology outpatient to undergo an elective nuclear stress test versus coronary catheterization in the outpatient setting.

Two weeks following discharge, the patient was seen by her cardiologist who reported she was at her baseline state of health, with complete resolution of presenting symptoms. She underwent elective left heart catheterization which demonstrated improved cardiac function, with normalization of LVEF to (55%). There were no wall motion abnormalities or apical ballooning. In addition, no evidence of significant coronary stenosis was seen, suggesting a diagnosis of TTC secondary to acute COVID-19 pneumonia. Repeat surveillance echocardiogram six months later again showed normal LVEF (55%), again with no visible wall motion abnormalities or apical ballooning. She was initiated on carvedilol and sacubitril-valsartan, with regularly scheduled outpatient cardiac follow-up.

## Discussion

Current NIH guidelines recommend that the management of COVID-19 pneumonia involves assessing the disease severity, underlying risk factors, and overall oxygen requirements. Important risk factors include age greater than 65 or age 50-64 with other comorbidities including cardiovascular or pulmonary chronic conditions, immunosuppression, underlying diabetes, and morbid obesity. For patients who are hospitalized but do not require supplemental oxygen, the NIH advises against using dexamethasone (AIIa) or other systemic corticosteroids (AIII). Patients hospitalized and requiring increased supplemental oxygen are recommended to receive dexamethasone (6 mg daily by mouth for up to 10 days) plus remdesivir (200 mg intravenously, followed by four days of 100 mg for a total of five days) (BIIa). Patients who are receiving dexamethasone but continue to have rapidly increasing oxygen requirements and systemic inflammation recommend adding baricitinib (BIIa) or tocilizumab (BIIa) to the current regimen. All patients without an indication for therapeutic anticoagulation should be given prophylactic heparin unless intake is contraindicated (AI; BIII for pregnant patients) [5].

The risk of developing TTC is known to occur in individuals under an increased amount of physical or emotional stress. Patients under increased stress, hormone dysregulation, and postmenopausal women have been found to have a higher risk of developing TTC than the normal population. During the COVID-19 pandemic, it was discovered that 7.75% of patients presenting with ACS were found to have underlying TTS. Prior to this, diagnosis in this population was around 1.5-1.8% [6]. It is hypothesized that TTC is secondary to an acute catecholamine surge following a physical or emotional stressor [7]. The catecholamine surge is believed to be responsible for direct toxicity resulting in myocardial dysfunction and stunning. In individuals who contract COVID-19, the autoimmune system releases increased cytokines which leads to an extensive systemic inflammatory response [8]. Elevated Inflammatory markers seen in the setting of COVID-19 include CRP, ESR, procalcitonin, IL-6, and ferritin [9]. The patient had an initial elevated CRP on admission but had resolved within seven days. An acute COVID-19 infection causes the release of these inflammatory markers, increased stress, and catecholamine surge which could lead to TTC as seen in our patient.

## Conclusions

Cardiac abnormalities such as arrhythmias, myocarditis, and acute heart failure have been associated with acute COVID-19 infections. Given the physiologic and emotional stress of an acute SARS-CoV-2 infection, TTC should be a consideration in patients with clear evidence of underlying cardiac dysfunction.
